# *Citrus leprosis virus C* Infection Results in Hypersensitive-Like Response, Suppression of the JA/ET Plant Defense Pathway and Promotion of the Colonization of Its Mite Vector

**DOI:** 10.3389/fpls.2016.01757

**Published:** 2016-11-25

**Authors:** Gabriella D. Arena, Pedro L. Ramos-González, Maria A. Nunes, Marcelo Ribeiro-Alves, Luis E. A. Camargo, Elliot W. Kitajima, Marcos A. Machado, Juliana Freitas-Astúa

**Affiliations:** ^1^Laboratório de Biotecnologia de Citros, Centro APTA Citros Sylvio Moreira, Instituto Agronômico de CampinasSão Paulo, Brazil; ^2^Escola Superior de Agricultura Luiz de Queiroz, Universidade de São PauloSão Paulo, Brazil; ^3^Universidade Estadual de CampinasSão Paulo, Brazil; ^4^Laboratório de Bioquímica Fitopatológica, Instituto BiológicoSão Paulo, Brazil; ^5^Instituto Nacional de Infectologia, Fundação Oswaldo CruzRio de Janeiro, Brazil; ^6^Embrapa Mandioca e FruticulturaCruz das Almas, Brazil

**Keywords:** cilevirus, herbivory, plant-virus-vector interaction, *Brevipalpus* mites, Arabidopsis, *Citrus sinensis*, hormonal crosstalk, RNA silencing

## Abstract

Leprosis is a serious disease of citrus caused by *Citrus leprosis virus C* (CiLV-C, genus *Cilevirus*) whose transmission is mediated by false spider mites of the genus *Brevipalpus*. CiLV-C infection does not systemically spread in any of its known host plants, thus remaining restricted to local lesions around the feeding sites of viruliferous mites. To get insight into this unusual pathosystem, we evaluated the expression profiles of genes involved in defense mechanisms of *Arabidopsis thaliana* and *Citrus sinensis* upon infestation with non-viruliferous and viruliferous mites by using reverse-transcription qPCR. These results were analyzed together with the production of reactive oxygen species (ROS) and the appearance of dead cells as assessed by histochemical assays. After interaction with non-viruliferous mites, plants locally accumulated ROS and triggered the salicylic acid (SA) and jasmonate/ethylene (JA/ET) pathways. ERF branch of the JA/ET pathways was highly activated. In contrast, JA pathway genes were markedly suppressed upon the CiLV-C infection mediated by viruliferous mites. Viral infection also intensified the ROS burst and cell death, and enhanced the expression of genes involved in the RNA silencing mechanism and SA pathway. After 13 days of infestation of two sets of Arabidopsis plants with non-viruliferous and viruliferous mites, the number of mites in the CiLV-C infected Arabidopsis plants was significantly higher than in those infested with the non-viruliferous ones. Oviposition of the viruliferous mites occurred preferentially in the CiLV-C infected leaves. Based on these results, we postulated the first model of plant/*Brevipalpus* mite/cilevirus interaction in which cells surrounding the feeding sites of viruliferous mites typify the outcome of a hypersensitive-like response, whereas viral infection induces changes in the behavior of its vector.

## Introduction

Most of the known plant viruses systemically infect their main plant hosts. Distinctively, *Citrus leprosis virus C* (CiLV-C), the causal agent of citrus leprosis, is unable to move long distances within any of its almost 50 natural or experimental host species belonging to at least 28 distant plant families (León et al., 2008; Nunes et al., 2012a,b; Arena et al., 2013; Garita et al., 2013, 2014). CiLV-C infection invariably produces chlorotic or necrotic lesions around the feeding sites of its mite vector. However, albeit it shows localized symptoms, citrus leprosis threatens citrus production in the Americas (Roy et al., 2015). Indeed, it is regarded as the most important viral disease affecting citrus in Brazil, the leading sweet orange producer in the world (Bastianel et al., 2010).

CiLV-C is the type member of the genus *Cilevirus* (Locali-Fabris et al., 2011). Virions are enveloped rod-like particles with 50–55 × 120–130 nm and its genome consists of two positive (+) sense single-stranded RNA molecules that contain 5′ cap structures and 3′ poly(A) tails. ORFs in the RNA1 (8745 nt) encode the RNA-dependent RNA polymerase and the putative 29 kDa coat protein. RNA2 contains four ORFs encoding the putative movement protein (MP), which shows conserved motifs of the plant virus MPs of the 30K superfamily, and the P15, P61, and P24 proteins with unknown functions (Locali-Fabris et al., 2006; Pascon et al., 2006). However, P61 and P24 display distant homology with structural proteins of the insect-specific negeviruses (Kuchibhatla et al., 2014).

False spider mites of the genus *Brevipalpus* (Acari: *Tenuipalpidae*) are polyphagous and cosmopolitan pests that colonize, in addition to citrus, several economically important crops and ornamentals plants (Childers et al., 2003a; Kitajima et al., 2010). In order to feed, false spider mites pierce and conceivably inject saliva into the plant mesophyll cells using their interlocked stylet, and after withdrawing it, they suck out the overflowed cell content of punctuated cells through the preoral cavity (Alberti and Kitajima, 2014). *Brevipalpus* spp. are haploid during their entire life-cycle, reproduce through thelytokous parthenogenesis and their adult populations are essentially females due to the presence of the endosymbiont bacterium *Cardinium* sp. (Weeks et al., 2001). Within the group of *Brevipalpus* species that transmit plant viruses, *B. yothersi* (synonym *B. phoenicis* Geijskes citrus type) is the main vector of the cileviruses (Beard et al., 2015; Sánchez-Velázquez et al., 2015; Ramos-González et al., 2016). Once acquired during any of the active phases of these mites, CiLV-C is persistently transmitted to distant parts within the same plant and to new ones, but not to their offspring (Bastianel et al., 2010; Kitajima and Alberti, 2014). CiLV-C replication in *B. yothersi* has been suggested (Roy et al., 2015).

Plants are recurrently invaded by attackers with distinct infecting or feeding strategies. Upon detection of the attacker-associated ligands, i.e., pathogen-associated molecular patterns (PAMP) and/or effectors, the plant immune system triggers a spectrum of dynamic responses to arrest the colonization process (Thomma et al., 2011; Cui et al., 2015). Phytohormones such as salicylic acid (SA), jasmonic acid (JA) and ethylene (ET) mediate a transcriptional reprogramming, tuning up defense responses that are modulated by the nature of the injury (Arimura et al., 2011; Pieterse et al., 2012; Alazem and Lin, 2015). The SA signaling pathway is primarily induced in response to biotrophic pathogens and piercing-sucking herbivores that cause minimal tissue damage. The JA pathway is subdivided in two antagonistic and interconnected branches that are activated in response to distinct stimuli. The ethylene responsive factor-branch (ERF-branch) is regulated by ERF transcription factors and synergistically cross-communicates with the ET pathway in response to necrotrophic invaders. On the contrary, MYC-branch, controlled by MYC transcription factors, is independent of ET and mediates defense against herbivores whose action greatly disrupts tissue integrity. Typically, SA antagonizes the JA/ET pathways, a plant strategy to efficiently allocate its resources according to the nature of the attack (Pieterse et al., 2012).

Plant defense against viruses mainly involves the RNA silencing machinery (Mandadi and Scholthof, 2013; Pumplin and Voinnet, 2013). Double stranded RNA (dsRNA) replication intermediates or structured RNA genomes from viruses can be considered a special case of PAMP, i.e., VAMP (virus-associated molecular pattern). VAMPs are recognized by Dicer-like nucleases (DCL), which further process them into virus-derived siRNAs. A guide strand of these molecules and an AGO protein are assembled into the RNA-induced silencing complex (RISC), which direct specific silencing of the homologous viral genome (Parent et al., 2012). Albeit previously considered an independent mechanism of antiviral resistance, the current understanding is that RNA silencing is connected with the hormonal pathways through the SA- and JA-induced RNA-dependent RNA polymerase 1 protein (RDR1) (Hunter et al., 2013).

In addition to hormones, plant response also involves the generation of reactive oxygen species (ROS). ROS may act as signal transduction molecules in the host, as toxic compounds against the attackers or as a blocking agent to their colonization by promoting the reinforcement of the plant cell walls (Foyer and Noctor, 2013; Camejo et al., 2016; Gilroy et al., 2016). Moreover, ROS accumulation may lead to the activation of the hypersensitive response (HR) (Foyer and Noctor, 2005; Xia et al., 2015). ROS and SA pathways are interconnected and mutually enhanced, promoting a self-amplifying feedback loop that drives HR (Xia et al., 2015). Hypersensitive-like response can also be observed as an outcome of plant-attacker interactions where the typically involved effectors and ligands are missing, suggesting the participation of yet unknown host proteins (Li et al., 2010).

Plant defenses are constantly challenged, and even hijacked, by the mechanisms of virulence from evolved attackers. Hormonal cross-talking that usually occurs during the plant immune response can be wielded by the attackers for their own benefit (Pieterse et al., 2012). In contact with plants, some insect eggs induce high level of SA, leading to a strong reduction of the JA-mediated defenses and, hence, decreasing their interference on the future larva feeding (Little et al., 2007; Bruessow et al., 2010; Gouhier-Darimont et al., 2013; Hilfiker et al., 2014). Extracts from these eggs reduce the MYC protein levels in a SA-dependent manner (Schmiesing et al., 2016). Moreover, insects and mites secrete proteins in their saliva which modulate, and even suppress, the plant defense response (Kant et al., 2015; Villarroel et al., 2016). In the saliva, proteins from arthropod-associated microorganisms, i.e., endosymbiont bacteria and viruses, are also responsible for plant defense elicitation (Chaudhary et al., 2014; Jaouannet et al., 2015). Similarly, plant viruses encode suppressors to efficiently overcome the RNA silencing (Li and Ding, 2006). Virus derived proteins have also been implicated in mutual benefit to pathogens and their vectors (Casteel and Falk, 2016).

Despite the uniqueness of the citrus leprosis pathosystem, its molecular aspects are poorly understood. To extend our comprehension of the mechanisms underlying it, the current study evaluated the model plant *Arabidopsis thaliana* by profiling the mRNA expression of marker genes of the main defense pathways during the course of its interaction with both non-viruliferous and CiLV-C viruliferous *Brevipalpus* mites using RT-qPCR. Moreover, we searched for evidence of an HR by performing histological tests on the injured plant tissues. Finally, we evaluated the feasibility of using Arabidopsis as a model host for citrus leprosis studies by comparing the transcript profiles of some selected Arabidopsis genes with their homologs of *Citrus sinensis*. In addition to the first evidence on how plants respond to both *Brevipalpus* feeding and CiLV-C infection, we present evidence of changes in the behavior of the vector mites in CiLV-C infected plants. Additionally, we provide data about CiLV-C inoculation efficiency by the mite and indicate the plant reference genes to be used in the current and future transcriptomic studies involving the citrus leprosis pathosystem.

## Results

### An inoculation access period of 6 h results in 100% of CiLV-C-infected arabidopsis plants by viruliferous *Brevipalpus yothersi* mites

**Figure 1:**
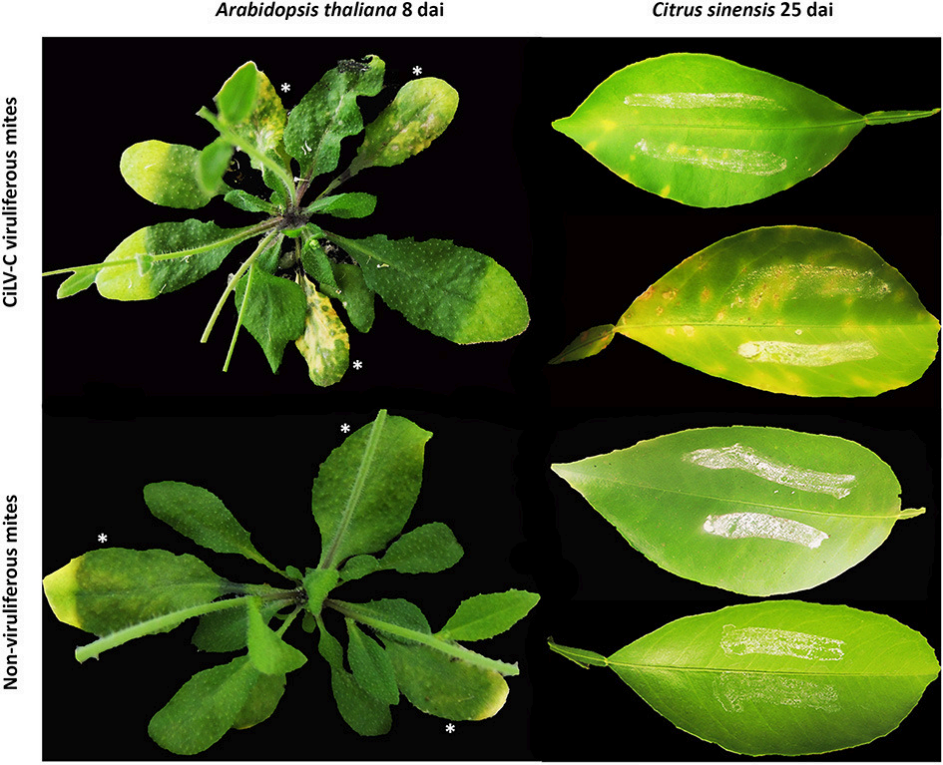
**Phenotypes of *Arabidopsis thaliana* (left)** and *Citrus sinensis* **(right)** plants infested with non-viruliferous and CiLV-C viruliferous *Brevipalpus yothersi* mites during the time course experiments. Pictures from Arabidopsis and *C. sinensis* were taken at 8 and 25 days after infestation, respectively. Infested leaves are indicated by asterisks (^*^).

Since the successful inoculation of CiLV-C is only achieved using viruliferous mites, the analysis of the transcriptional profiles of plant genes involved in the interaction with this virus can only be inferred by comparing the profiles of plants infested with viruliferous and non-viruliferous mites. However, as upon infestation the mites do not feed in a synchronous manner, the time of CiLV-C inoculation may vary among a set of infested plants. To reduce the impact of phased out infections over the analysis of the expression profiles of genes implicated in the earlier steps of the interaction, we conducted a preliminary experiment to determine the minimal feeding period necessary to achieve 100% of infection of plants infested with viruliferous mites. Seven to ten days after infestation (dai), Arabidopsis plants inoculated with viruliferous mites either for 4 or 6 h showed the typical symptoms of CiLV-C infection; i.e., chlorotic spots in green leaves and green islands in yellow senescent ones (Arena et al., 2013) (symptoms as in Figure 1). However, the number of symptomatic plants differed between the two treatments. In the 4 h treatment only 30% of the plants displayed symptoms compared to 100% in the 6 h treatment (Table 1). Presence of CiLV-C in all symptomatic plants was confirmed by RT-PCR (data not shown). Thus, from these analyses we determined that 6 h was an appropriate time to initiate the evaluation of the Arabidopsis responses to CiLV-C infection.

**Table 1:** **CiLV-C inoculation efficiency of *Arabidopsis thaliana* with five viruliferous *Brevipalpus* mites after different inoculation access periods (IAP)**.

**IAP**	**Test-plants (n)**	**Symptomatic plants (n)**	**PCR-positive plants (n)^b^**	**Inoculation efficiency (%)**
4 h	10	3	3	30
6 h	10	10	10	100
Control^a^	10	10	10	100

a Inoculation positive control, where CiLV-C viruliferous mites were kept onto plants throughout the experiment.

b Plants were evaluated by RT-PCR using primer pairs designed for a region within the CiLV-C MP gene (Locali et al., 2003).

### *F-BOX, SAND* and *TIP41* are suitable reference genes for transcript normalization during arabidopsis/mite/cilevirus interaction

A time course experiment to evaluate Arabidopsis interaction with non-viruliferous and viruliferous mites was set up. Six candidate reference genes (*EF1A, F-BOX, GAPDH, PPR, SAND*, and *TIP41*) for transcript normalization were evaluated by assessing their expression levels. Quantification cycle (Cq)-values ranged from 23.98 to 36.01 (Table 2), and only those whose transcripts accumulated in moderate quantities (15 < Cq < 30, Wan et al., 2010) were further selected (i.e., *EF1A, F-BOX, GAPDH, SAND*, and *TIP41*). Statistical analysis using geNorm (Vandesompele et al., 2002) revealed that the *F-BOX, SAND*, and *TIP41* genes presented the lowest *M*-values, which correspond to the highest expression stabilities. Since data normalization is preferably done using at least three reference genes (Vandesompele et al., 2002), these were selected for the RT-qPCR analysis conducted in this work.

**Table 2:** ***Arabidopsis thaliana* candidate reference genes ranked according to their expression stability**.

**Gene**	**Locus**	**Mean Cq (±*SD*)^a^**	**Stability value (*M*)^b^**	**Ranking^b^**
*FBOX*	AT5G15710	30.46 ± 0.29	0.18	1
*TIP41*	AT4G34270	28.15 ± 0.31	0.18	1
*SAND*	AT2G28390	28.30 ± 0.26	0.19	2
*EF1α*	AT5G60390	23.98 ± 0.38	0.22	3
*GAPDH*	AT1G13440	30.19 ± 0.71	0.34	4
*PPR*^c^	AT5G55840	36.01 ± 0.41	-	-

a Quantification cycle (Cq) and standard deviation (SD) values were obtained from RT-qPCR of 27 samples (three technical replicates of nine biological samples across three experimental conditions: plants infested with viruliferous mites, non-viruliferous mites or not infested).

b M-values, calculated by geNorm (Vandesompele et al., 2002) based on RT-qPCR data, increase from the most stable pair of genes to the least stable.

c PPR gene was excluded from analyses due to its low expression level (Cq-value > 35).

### Infestation with non-viruliferous mites induces the SA pathway and a response against necrotrophic rather than herbivorous attackers in arabidopsis

**Figure 2:**
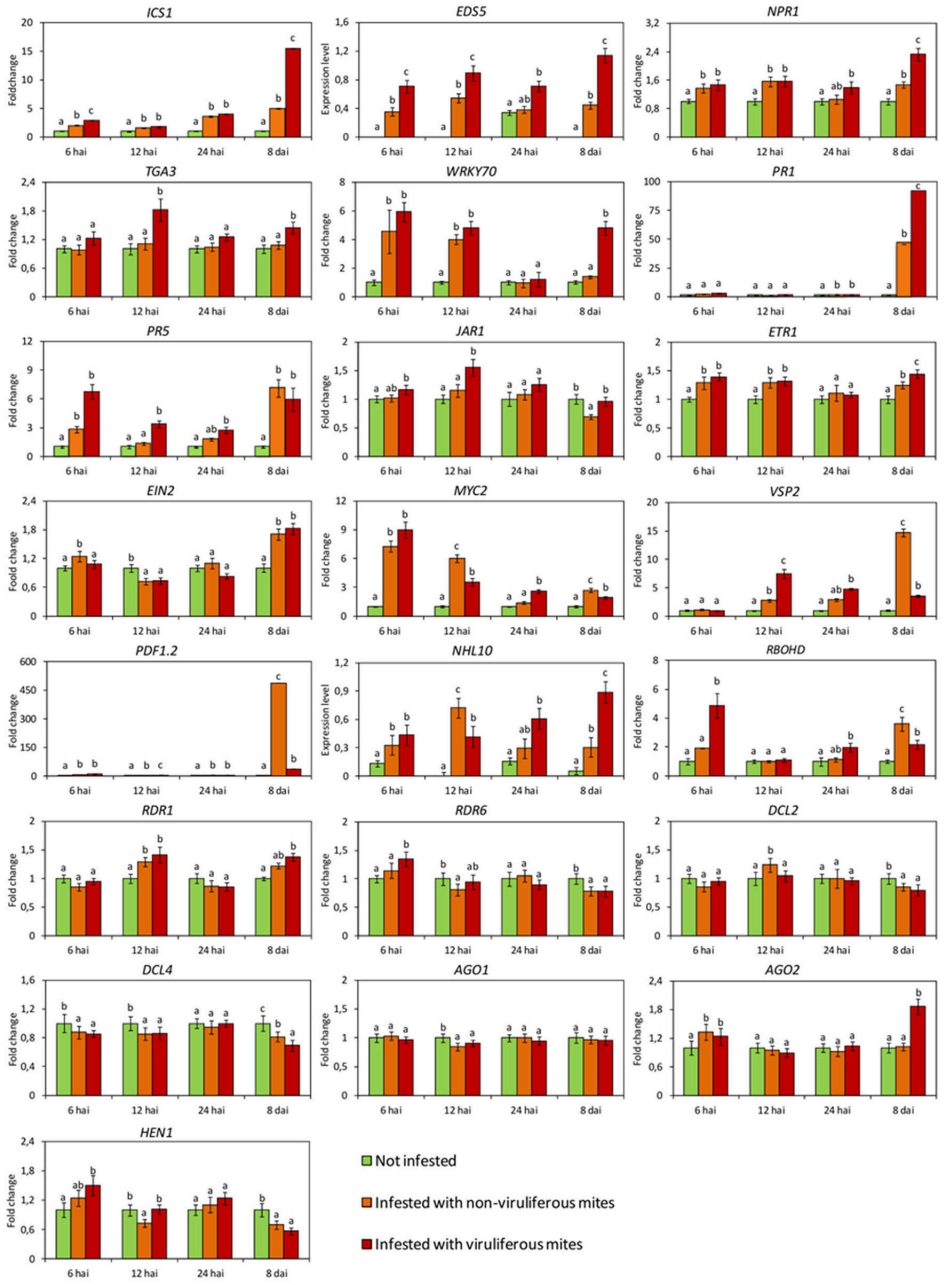
**Expression profiles of *Arabidopsis thaliana* genes involved in the main plant defense pathways assessed by RT-qPCR**. In green, plants kept without mites. In orange, plants infested with non-viruliferous mites. In red, plants infested with CiLV-C viruliferous mites. Data are presented as fold change values in comparison with not infested plants (with fold change value set to 1) or expression levels when transcripts were not detected in not infested plants. Values represent the mean of 10 biological replicates for each set. Error bars represent standard errors. Different letters correspond to different expression levels between treatments within the same time point (ANOVA and Tukey's HSD *post-hoc* test, α < 0.05). hai, hours after infestation; dai, days after infestation.

Expression of several genes involved in SA response (*ICS1, EDS5, NPR1, WRKY70* and *PR5*), JA/ET pathways (*ETR1, EIN2, MYC2*, and *PDF1.2*) and gene silencing mechanism (*AGO2*), in addition to marker genes for HR (*NHL10*) were induced as soon as at 6 h after infestation (hai) (Figure 2), indicating a rapid and combined response of the plants to the mites.

Genes of the SA pathway remained induced throughout the time course, i.e., *ICS1*: encoding the enzyme responsible for main SA biosynthesis in defense responses, *EDS5:* the transporter of SA from the chloroplast to cytoplasm, *NPR1*: the main regulator of SA-responsive genes and the transcription factor (TF) *WRKY70*. While statistical analysis indicated an induction of the defense gene *PR5* as early as 6 hai, the expression of this gene as well as that of *PR1* were noticeably up-regulated at 8 dai (Figure 2).

The expression of most of the evaluated genes of the JA/ET pathways was also induced at least at one time point with the exception of *JAR1*, whose relative expression was not altered at the earlier times of the infestation and was reduced at 8 dai (Figure 2). Up-regulation of the ethylene receptor gene (*ETR1*) was observed at 6, 12 hai and 8 dai; while induction of the main positive regulator downstream of the ethylene perception (*EIN2*) was detected at 6 hai and 8 dai. Up-regulation of the TF *MYC2* was detected at 6 hai, but its relative expression was gradually reduced at the following two time points and increased again at 8 dai. The relative expression of the genes *PDF1.2* and *VSP2* increased during the interaction, although at different levels and time points. Induction of *PDF1.2* was noteworthy since its transcript levels were 500-fold higher than in not infested plants (Figure 2).

Expression of genes of the RNA silencing mechanism was also modulated in response to mite feeding (Figure 2). Induction was verified for *RDR1* (12 hai), *AGO2* (6 hai) and *DCL2* (12 hai). We also observed repression of *AGO1* at 12 hai, of *HEN1* and *RDR6* at 12 hai and 8 dai, and of *DCL4* at 6, 12 hai and 8 dpi.

*RBOHD* gene, which encodes a NADPH oxidase enzyme responsible for ROS production, was highly induced at 8 dai, whereas the HR specific gene *NHL10* was found up-regulated throughout the time points assessed, except at 24 hai (Figure 2).

### Arabidopsis infestation with CiLV-C viruliferous *B. yothersi* mites enhances SA response and suppresses the JA/ET pathway

**Figure 3:**
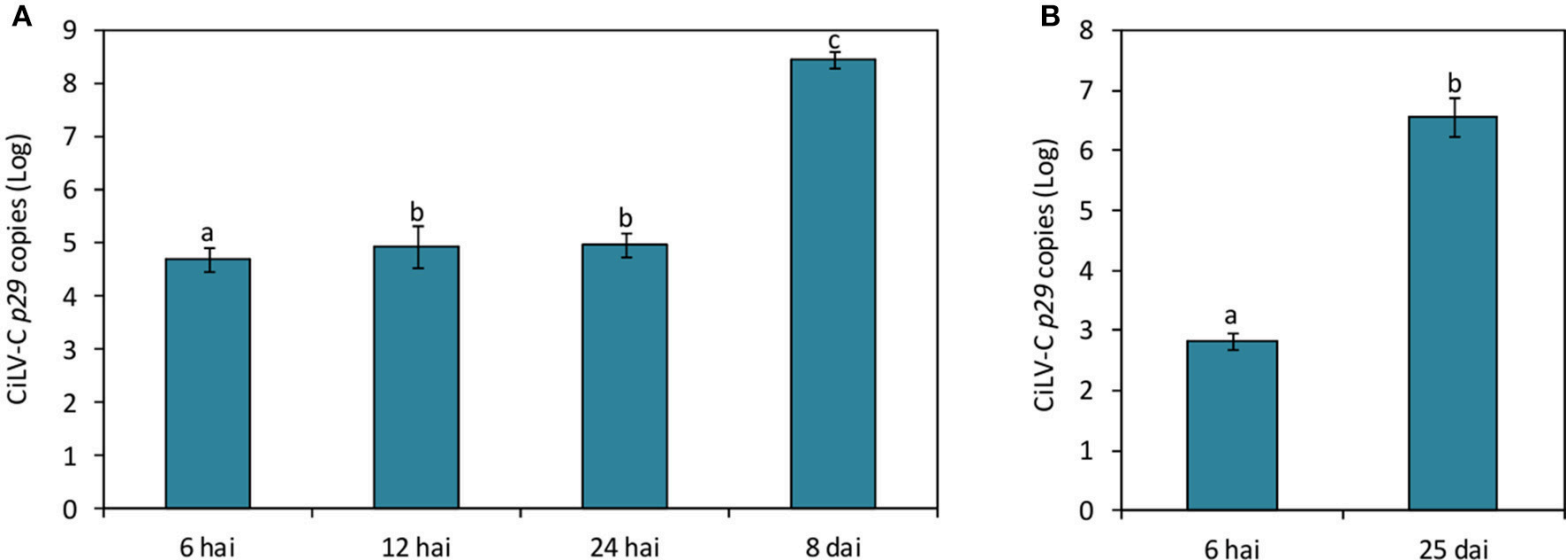
**CiLV-C loads on *Arabidopsis thaliana* (A)** and *Citrus sinensis* **(B)** plants infested with viruliferous *Brevipalpus yothersi* mites throughout the time course of the interaction. Data are presented as absolute number of copies (log) of the CiLV-C *p29* gene normalized against *SAND* genes corresponding to each plant species. Values represent the means of 10 biological replicates for each time point. Standard errors are represented on each bars. Different letters correspond to different number of copies between the time points assessed (ANOVA and Tukey's HSD *post-hoc* test, α < 0.05).

CiLV-C loads during the time course experiment were assessed using a RT-qPCR assay established during this work. Virus was detected in all plants infested with viruliferous mites, whereas its absence was confirmed in plants either infested with non-viruliferous mites or non-infested. In the subset of plants collected at 12 and 24 hai, virus titers were ca. 2-fold higher (*p* < 0.05) than in plants collected at 6 hai, whereas at the time of symptom appearance (8 dai), the viral load increased 2000-fold (Figure 3A).

Overall, the expression of the SA pathway related genes were higher in plants infested with viruliferous mites than in those infested with non-viruliferous ones (Figure 2). For instance, the expression of genes coding for proteins acting upstream of the pathway (*ICS1* and *EDS5*) was triggered in higher levels as early as 6 hai, whereas those encoding downstream proteins were gradually up-regulated. Induction of the TF *TGA3* and the defense protein *PR5* were observed in samples collected at 12 hai. In the case of *WRKY70, NPR1*, and *PR1*, maximum relative expressions were detected at the latest stage of the infection (8 dai), when the highest viral loads were reached (Figure 3A).

In contrast to the general activation of genes of the SA pathway, genes of the JA/ET pathways displayed distinct expression patterns (Figure 2). Upon infection, genes encoding the receptors for JA (*JAR1*) and ET (*ETR1*) were up-regulated, although the expression levels of downstream genes of this pathway were reduced. *EIN2* and *MYC2* were repressed at 6 and 12 hai, respectively, whereas the relative expression of the pathway outcome genes *PDF1.2* and *VSP2* showed a marked reduction at the end of the evaluation.

Core genes of the RNA silencing mechanism showed distinct expression profiles in plants infested with non-viruliferous compared to those infested with viruliferous mites (Figure 2). *RDR6* and *HEN1* were induced earlier (6 and 12 hai, respectively) than *AGO2* (8 dai), whereas the Dicer-like nucleases genes were suppressed during the infection. The lowest relative expressions of *DCL2* and *DCL4* were detected at 12 hai and 8 dai, respectively.

The expression patterns of the marker genes of the ROS burst and HR were up-regulated in response to virus infection. Induction of *RBOHD* was observed as early as 6 hai, whereas induction of *NHL10* was detected at 8 dai.

### Sweet orange plant response to citrus leprosis mirrors major hallmarks of the arabidopsis/*Brevipalpus* mite/CiLV-C interaction

The expression profiles of some key genes involved in the defense response of Arabidopsis to *Brevipalpus* mites and CiLV-C (*PR1, MYC2, AGO2*, and *WRKY70*) were also evaluated in infested sweet orange plants.

In citrus plants infested with viruliferous mites, the virus was detected by RT-qPCR as early as 6 hai (Figure 3B). Viral loads increased around 10^4^-fold after 25 days of infection. Symptoms were firstly observed at 17 dai, when small chlorotic spots started to develop in some plants. Typical chlorotic and necrotic lesions of citrus leprosis only were observed at 25 dai (Figure 1).

**Figure 4:**
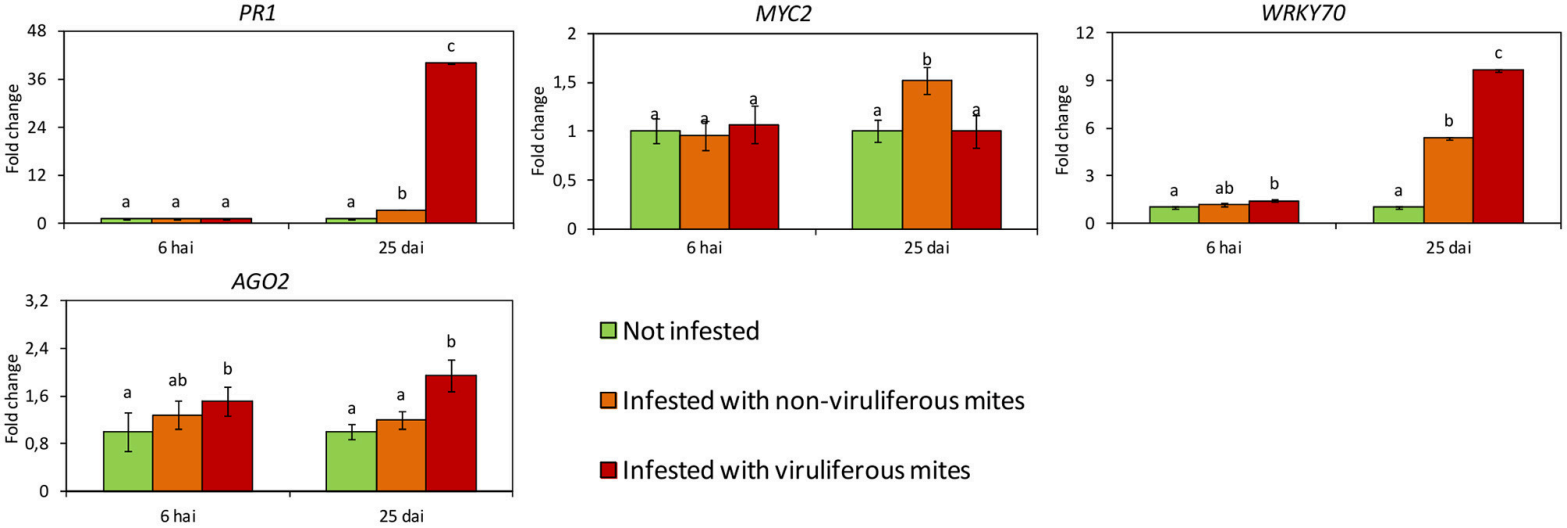
**Expression profiles of *Citrus sinensis* genes involved in the main plant defense pathways assessed by RT-qPCR**. In green, plants kept without mites. In orange, plants infested with non-viruliferous mites. In red, plants infested with CiLV-C viruliferous mites. Data are presented as fold change values in comparison with not infested plants (with fold change value set to 1). Values represent the mean of 20 biological replicates for each set. Error bars represent standard errors. Different letters correspond to different expression levels between treatments within the same time point (ANOVA and Tukey's HSD *post-hoc* test, α < 0.05). hai, hours after infestation; dai, days after infestation.

Differences in gene expression were not observed between not infested plants and those infested with non-viruliferous mites at 6 hai. At this time, only the expression of *WRK70* and *AGO2* were induced in response to the infestation with viruliferous mites (Figure 4). However, at 25 dai all genes had their expression altered. Particularly, high relative expression of *AGO2* was observed on plants infested with viruliferous mites, suggesting a RNA silencing response specific to CiLV-C infection. The SA-dependent *PR1* gene was induced both in plants infested with non-viruliferous and viruliferous mites, but in the latter treatment its relative expression was much higher, probably indicating an exacerbated response to the viral infection progress. By contrast, the expression of the JA/ET-related TF *MYC2* was induced only in plants infested with non-viruliferous mites, suggesting that its expression is reduced to basal levels upon infection by CiLV-C. The expression of *WRKY70*, the TF responsible for the SA-JA cross-talk, was induced as a result of mite feeding, but it was higher in plants infected with CiLV-C.

### CiLV-C infection intensifies the oxidative stress and cell death in arabidopsis plants

**Figure 5:**
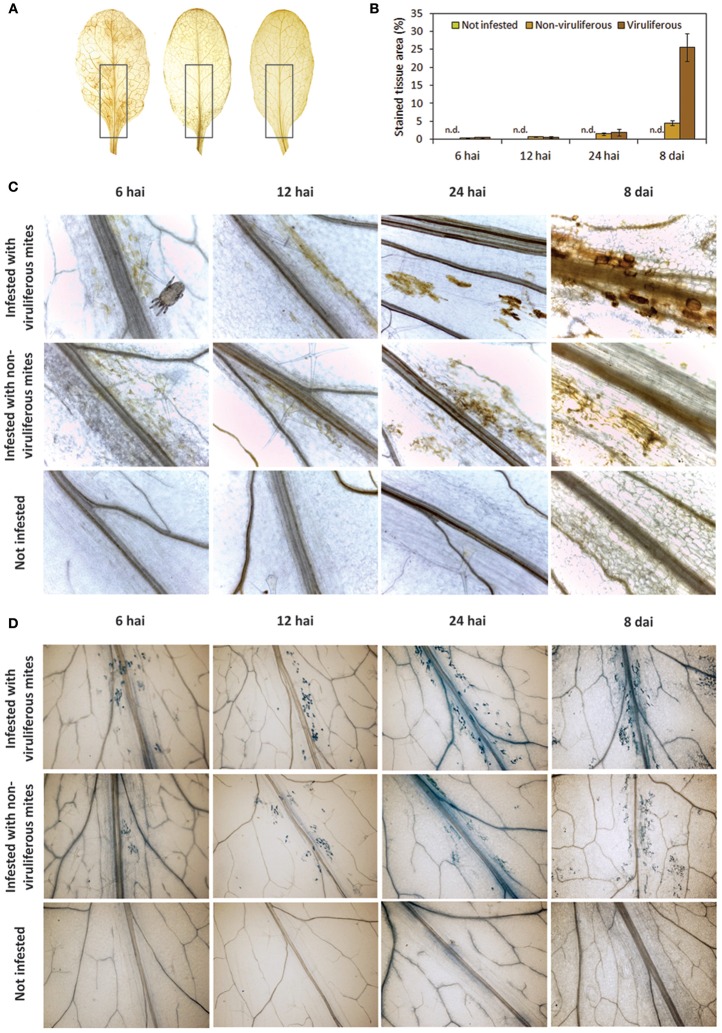
**Accumulation of reactive oxygen species (ROS) and cell death in *Arabidopsis thaliana* plants infested with non-viruliferous and CiLV-C viruliferous *Brevipalpus yothersi* mites at 6 hours after the infestation (hai), 12 hai, 24 hai, and 8 days after the infestation (dai). (A)** Detection of hydrogen peroxide by 3,3′-diaminobenzidine (DAB) staining at 8 dai on leaves infested with viruliferous mites, non-viruliferous mites and not infested, respectively. Highlights on leaves midribs represent the regions where mites concentrate, used to quantification of stained area. **(B)** Quantitative measure of DAB brownish areas on highlighted regions along the course of the infestation. Values represent the means of 10 biological replicates for each set. Error bars represent standard errors. **(C)** Detail of ROS detection in leaves stained with DAB. **(D)** Detail of cell death in leaves stained with trypan blue.

ROS production was detected using the assay based on the oxidation of 3,3′-diaminobenzidine (DAB), which turns brown in the presence of hydrogen peroxide (H_2_O_2_). Discrete brownish spots were detected in leaves collected as early as 6 and 12 hai in plants of both mite-infested treatments, and their frequency increased at 24 hai comprising up to 1.5 and 1.9% of the stained tissue area in plants infested with non-viruliferous and viruliferous mites, respectively (Figures 5A,B). DAB stained area concentrated in the leaf midribs, which correspond to preferred mites feeding regions. At 8 dai, the number of spots increased and they were observed alongside the leaves as well (Figure 5C). Brownish areas were noticeably larger and more frequently seen in virus-infected leaves, representing approximately 25.6% of the leaf area. In plants infested with non-viruliferous mites the stained tissue only represented 4.5% of the leaf. H_2_O_2_ production was not observed in non-infested plants.

In infested plants, dead cells were detected since 6 hai and they increased in number over time as revealed by trypan blue dying (Figure 5D). Foci of dead cells were mostly confined to the leaf main veins and chiefly corresponded to individual cells during the early times of the interaction (6–24 hai). Leaves of plants infested with viruliferous mites presented a higher frequency of blue spots and these were larger as they likely involved greater number of cells. As observed in the accumulation of H_2_O_2_, spots of dead cells were scattered throughout the virus-infected leaves at 8 dai. No stain was detected in leaves of the healthy control plants.

### CiLV-C infection in arabidopsis modules the behavior of *Brevipalpus* mites

**Figure 6:**
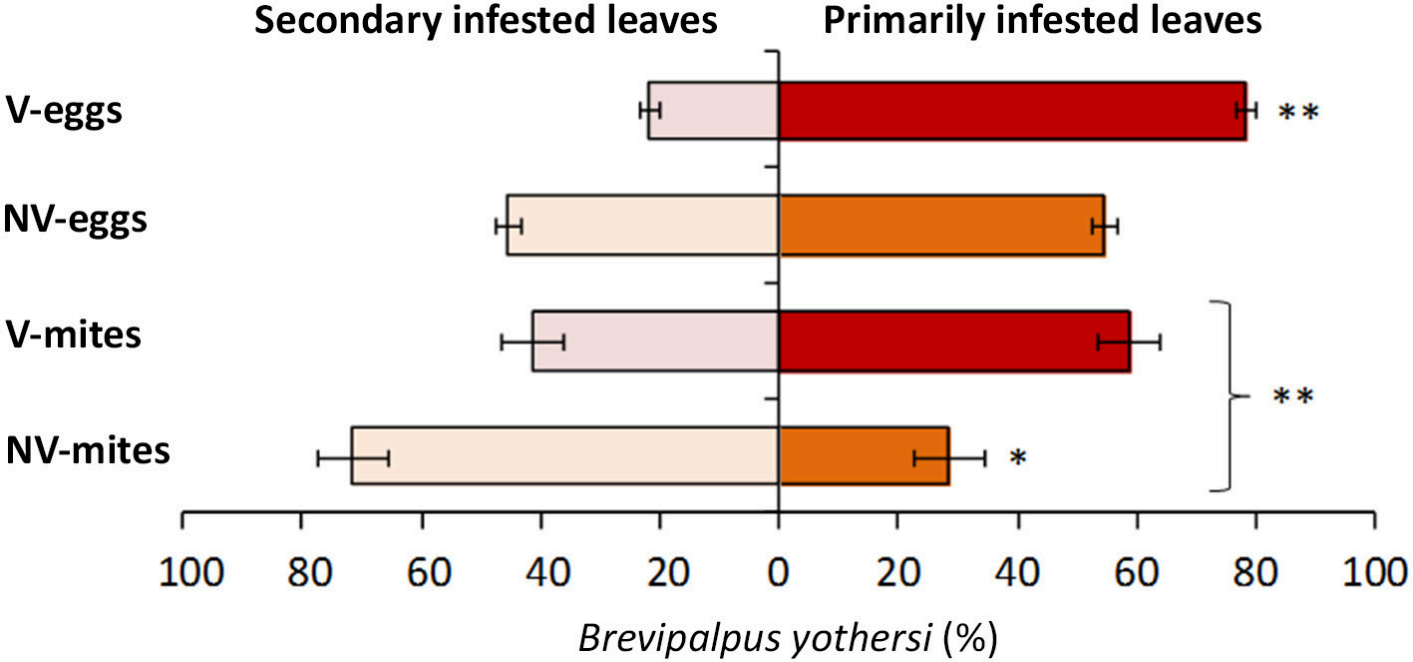
**Distribution of *Brevipalpus yothersi* adult mites and eggs in *Arabidopsis thaliana***. Non viruliferous (NV) or CiLV-C viruliferous (V) mites were deposited in three leaves of each plant and were counted at 13 days after the infestation. Data are presented as the average percentages of mites or eggs in the primarily infested leaves (where mites were initially deposited) and secondary infested leaves (due to mite migration to contiguous leaves). Error bars represent standard errors. Statistically significant differences at *p* < 0.05 (^*^) or < 0.01 (^**^) are indicated. Bracket indicates the comparison between the number of NV and V mites.

To evaluate the putative role of CiLV-C infection over the behavior of *Brevipalpus* mites, two sets of healthy Arabidopsis plants were infested with two populations of either viruliferous or non-viruliferous adult mites. Typical localized symptoms started to appear at 7 days after infestation with viruliferous mites only. After 13 days of infestation, the number of mites per plant was higher in those infested with viruliferous ones (*p* < 0.01) (Supplementary Table 2, Figure 6). While no significant differences in mite numbers were observed between primarily and secondary infested leaves in plants that received viruliferous mites (*p* > 0.05), the non-viruliferous mites were unevenly distributed throughout the infested plants, with higher population densities found in the secondary infested leaves (*p* < 0.05). The total number of mite eggs counted in the two sets of plants did not differ significantly (*p* > 0.05). However, the number of eggs in the primarily infested leaves was superior to that in the secondary infested leaves (*p* < 0.01) for those plants infested with viruliferous mites, whereas no such differences were observed for plants infested with non-viruliferous mites (*p* > 0.05).

## Discussion

In this work we provide insights into plant response during citrus leprosis infection, an emergent viral disease that threatens citrus production in the Americas (Roy et al., 2015). Remarkably, this disease reveals an atypical pathosystem in which its etiological agent, CiLV-C, does not systemically spread in the host plants and half of the genes encoded by its genome are considered as orphans since they have no homologs in other viral species (Locali-Fabris et al., 2006; Tautz and Domazet-Lošo, 2011). Additionally, *Brevipalpus yothersi* mites, the CiLV-C vector, also attack hundreds of plant species of very distinct families, have worldwide distribution and exhibit an unusual biology (Weeks et al., 2001; Childers et al., 2003b).

To shed light on citrus leprosis pathosystem, we evaluated the transcriptional profiles of marker genes of Arabidopsis defense pathways, ROS production and the occurrence of cell death upon infestation with either non-viruliferous or CiLV-C viruliferous mites. Moreover, we extended these analyses to sweet orange in order to validate the use of Arabidopsis as a model plant for the study of citrus leprosis.

As a result of the interaction of Arabidopsis with non-viruliferous mites, small areas likely involving a few dead cells proliferated in infested leaves while increased amounts of H_2_O_2_ were detected within early stages of the interaction. Such a pattern of dead cell patches may be a direct consequence of mite feeding and resembles that observed during interaction of the mite *Tetranychus urticae* with Arabidopsis and bean plants in which only one cell is targeted by the mite's stylet (Bensoussan et al., 2016). *Brevipalpus* activity also elicited the expression of SA pathway genes (biosynthesis, signaling and response), and the JA/ET responsive genes *PDF1.2* and *VSP2.* Notably, the expression level of *PDF1.2* was much higher than that of *VSP2*, ca. 500- and 15-fold, respectively, in comparison to the not infested treatment at 8 dai.

The switching between strong and mild expression levels of the JA-responsive genes was previously described as an herbivore strategy to rewire the plant response in its favor (Verhage et al., 2011). Based on this, our data supports the hypothesis that *B. yothersi* mites might manipulate plant defenses to their own benefit. Typically, plant response to herbivory and tissue damage triggers the JA signaling pathway, which controls two major acting branches (Wu and Baldwin, 2010; Arimura et al., 2011; Erb et al., 2012). Activation of each branch is mediated by several TFs usually represented by MYC2 and ERF1. Whereas the ERF-branch induces the expression of genes such as *PDF1.2* to counteract necrotrophic pathogens, the MYC-branch up-regulates genes such as *VSP2*, which encodes a phosphatase with anti-herbivory activity (Verhage et al., 2011; Kazan and Manners, 2013). TFs from the MYC-branch also interact with TFs of the ERF-branch, and vice versa, repressing each other (Song et al., 2014). Under herbivory attack, wild-type Arabidopsis plants preferably activate the MYC branch; however, activation of the ERF-branch occurs, for instance, in plants impaired in the MYC-branch, as in the case of Arabidopsis *jar1-1* mutants (Verhage et al., 2011). In the Arabidopsis-*Brevipalpus* interaction assessed in this study, the down-regulation of *JAR1* was accompanied by an increased expression of *PDF1.2* detected at the end of the time course. Therefore, repression of *JAR1* could redirect the JA pathway toward *PDF1.2* thus reducing the anti-herbivory defense conferred by the MYC-branch.

Arabidopsis infestation with *Brevipalpus* also triggered SA signaling leading to the up-regulation of *PR* responsive genes within 24 hai. This has been observed in responses to certain arthropods that cause mild tissue injuries such as the piercing-sucking insects like aphids (Zarate et al., 2007; Arimura et al., 2011). By exploiting the natural cross-talk between the SA-JA/ET signaling pathways, these herbivores suppress JA mediated defenses favoring their own performance (Zarate et al., 2007; Hogenhout and Bos, 2011; Zhang et al., 2013). When induced, SA down-regulates the transcription of JA responsive genes from both ERF and MYC branches through a mechanism mediated by NPR1 and WRKY70 (Caarls et al., 2015). However, differently than in insects, in plant-mite interactions, both SA and JA/ET signaling pathways are simultaneously induced and apparently do not antagonize each other (Zhurov et al., 2014). Although this could be the case of *Brevipalpus*-Arabidopsis interaction, where both *VSP2* and *PDF1.2* were induced at most of the analyzed time points, we cannot exclude that during this interaction SA and JA may actually antagonize each other, although partially, and the JA response we report here is intermediate. Such a relationship was also suggested during plant interaction with *Tetranychus* mites (Alba et al., 2015).

Modulation of plant response by herbivores has been shown to occur by effector proteins present in their saliva (Hogenhout and Bos, 2011). Piercing-sucking insects puncture and deliver effectors to suppress plant defenses and establish compatible interactions. Application of oral secretions of *Pieris rapae* caterpillars on Arabidopsis leaves, for example, activates the ERF-branch, suggesting that compounds in the saliva divert plant response favoring the herbivore (Verhage et al., 2011). Analogous to aphids, spider mites also suppress plant defenses (Sarmento et al., 2011; Alba et al., 2015) through delivery of effectors via their saliva (Villarroel et al., 2016). *Tetranychus evansi* suppresses defense routes in tomato, reducing deterrent compounds to even lower levels than constitutive ones expressed in healthy plants (Sarmento et al., 2011). *Brevipalpus* feeding involves the piercing of plant tissues, and likely, the injection of saliva inside the host cells through a tube formed between its interlocked stylets (Alberti and Kitajima, 2014). Therefore, our results indicating the induction of signaling pathways associated to herbivore manipulation in Arabidopsis suggest that *Brevipalpus* might also inject effectors in their hosts through the saliva. Since herbivore host range is likely limited by its ability to suppress effectual plant defenses (Hogenhout and Bos, 2011), it is suggested that generalists are more suppressive of plant defense than specialists (Ali and Agrawal, 2012). *Brevipalpus* mites are extreme generalists, colonizing a wide range of hosts that includes more than 900 plant species spanning more than a 100 plant families (Childers et al., 2003b). In this regard, the repertoire of immune response suppression strategies observed during the interaction with Arabidopsis consistently supports the polyphagia of *Brevipalpus* mites.

CiLV-C infection in Arabidopsis triggered SA-dependent genes, a common response of plants to biotrophic pathogens (Pieterse et al., 2009). From a general point of view, the patterns of expression of the SA pathway genes resembled those observed after infestation with non-viruliferous mites, though fold changes of some genes such as *NPR1* and *WRKY70* were higher in the presence of the virus. This activation likely reaches the threshold required to trigger the down-regulation of JA/ET responsive genes. In the presence of CiLV-C, *PDF1.2* and *VSP2* were drastically repressed (13- and 4-fold lower, respectively) at latest stages of the infection when compared to plants infested with non-viruliferous mites, suggesting the occurrence of SA-JA antagonism.

Infestation of Arabidopsis with CiLV-C viruliferous mites typically caused viral infection in the leaves where mites were deposited (primarily infestation). Viral infection as consequence of a secondary infestation due to mite migration between contiguous leaves were barely detected at 8 dai, as observed in Figure 1, or even after 13 dai, when the final evaluation was performed. Results obtained in this work indicated that infected leaves become more attractive for the mites. In absence of viral infection, mites were preferentially found in the secondary infested leaves. This behavior seem to be logic considering that the area for secondary infestation is the largest, comprising more and newer leaves available for mite colonization. In contrast, in plants infected by viruliferous mites, no differences were observed in their distribution, and leaves showing typical CiLV-C localized symptoms harbored the largest number of eggs, indicating the preference for oviposition in infected leaves. Moreover, density of mites (mite/plant) reached higher values in the infected Arabidopsis plants. Interestingly, CiLV-C infected sweet oranges plants seem to be also beneficial for *Brevipalpus* mite population. In a multifactorial experiment addressed to evaluate the influence of citrus rootstocks on the relationship between the mite *Brevipalpus* sp. and citrus leprosis disease (Andrade et al., 2013), mite density was significantly higher in CiLV-C infected plants.

Recent studies suggest that some virus-infected plants are more attractive for the viral vector settlements and/or more beneficial to the vector development (Mauck et al., 2012; Casteel et al., 2014; Prager et al., 2015; Su et al., 2015; Casteel and Falk, 2016). Viruses that depend on vectors to move from infected to healthy host plants use this strategy as an effective mean to improve their transmissibility (Belliure et al., 2004; Abe et al., 2012). For instance, infection by *Tomato spotted wilt virus* (TSWV) induces the SA-mediated pathway, which decreases JA-regulated defenses leading to enhanced attractiveness of plants to its vector, the thrips *Frankliniella occidentalis* (Abe et al., 2012). SA pathway induction by CiLV-C and reduction of JA/ET mediated response reported here, in addition to other unknown mechanisms, may benefit *Brevipalpus* infestation. This issue is of special significance in the case of the conspicuous nature of the localized colonization of CiLV-C, because relationship with its vector is not only essential for plant to plant transmission, but also to the infection of other parts within the same plant. On the other hand, CiLV-C may act as a helper (effector-like) factor of mite infestation to suppress the plant defenses. However, whether the viral infection mediated by viruliferous mites indeed may help the mite fitness to plant colonization is yet unclear and has been addressed in ongoing experiments.

During Arabidopsis interaction with both non-viruliferous and viruliferous *Brevipalpus*, fold changes of core genes of the RNA silencing machinery showed low variation and the expression profiles were kept mostly invariable, except in the cases of *RDR6* and *AGO2*, whose expressions were enhanced in response to virus infection at 6 hai and 8 dai, respectively. RISC activity against plant viruses preferentially involves AGO1 and, in case of its suppression, the cell switches to a second layer of defense mediated by AGO2 (Harvey et al., 2011). Increased levels of *AGO2* during virus infection have been observed in Arabidopsis during *Potato virus X* (PVX), *Cucumber mosaic virus* (CMV), *Turnip crinkle virus* (TCV), *Tobacco rattle virus* (TRV), and *Turnip mosaic virus* (TuMV) infections (Carbonell and Carrington, 2015). Control of *AGO2* mRNA levels is mediated by the microRNA miR403 via AGO1 (Harvey et al., 2011). Inactivation of AGO1 by some virus suppressor of RNA silencing (VSRs), e.g., the p38 protein from TCV, leads to the expression of AGO2. TCV-p38 VSR activity is exerted by a WG motif that acts as a hook for AGO1 protein, disabling RISC (Azevedo et al., 2010). Interestingly, a WG/GW motif is also present in the COOH terminal domain of the CiLV-C RdRp protein, suggesting that the activation of AGO2 reported here could result from the hijacking of AGO1 in an analogous manner as the one described for TCV. Further experiments need to be carried out to test the putative VSR activity derived from CiLV-C proteins.

Plant gene expression during CiLV-C resembles that observed in the course of a plant-virus interaction, where SA pathway and RNA silencing are activated (Mandadi and Scholthof, 2013). The current understanding is that both defense responses act coordinately to counteract viral infection (Alamillo et al., 2006; Lewsey et al., 2010; Jovel et al., 2011; Hunter et al., 2013). SA-mediated defense interacts with RNA silencing through ICS1- and NPR1-dependent up-regulation of RDR1, which participates in the generation of secondary siRNA (Hunter et al., 2013). Evidence of interplay between both defenses against viral infections are increasing in the literature, e.g., *Plum pox virus* (PPV) infection of *Nicotiana tabacum* results in the SA-mediated potentiation of RNA silencing, thus inhibiting PPV systemic movement (Alamillo et al., 2006). In sour orange plants (*Citrus aurantium*), *Citrus tristeza virus* (CTV) accumulation and spread are enhanced when the genes *RDR1, NPR1*, or *DCL2-DCL4* are silenced (Gómez-Muñoz et al., 2016). For the CiLV-C/Arabidopsis interaction, the induction of *RDR1* corroborates with this mechanism and indicates that the early activation of SA in response to mite feeding could pre-induce resistance to CiLV-C replication.

As observed during the interaction of Arabidopsis with non-viruliferous *Brevipalpus*, infestation with CiLV-C viruliferous mites also elicited the production of H_2_O_2_. However, in this case both the intensity of histochemical signals and fold changes of *RBOHD* were higher than in the former case, further confirming our conclusion that infection by the virus amplifies the host responses. Moreover, in these plants, the frequency of patches of dead cells was also higher, indicating that the plant actively recognizes the presence of CiLV-C. The oxidative burst both activates cell death at the infection site during HR and signals defense pathways beyond the infection site (Levine et al., 1994; O'Brien et al., 2012). Thus, the observed cell death during CiLV-C infection may be a consequence of HR which on its turn would limit the systemic colonization of the host. Indeed, characteristic necrotic symptoms of CiLV-C infection resemble the outcome of HR-like, although the evolution up to necrosis shows an atypically slower progression in sweet orange than that expected in case of a typical hypersensitive reaction.

Induction of HR in cells of the vascular tissue of sweet orange leaves was previously proposed as the cause for the non-systemic spread of CiLV-C (Marques et al., 2007, 2010). In this regard, caution has to be taken to interpret these and our own results. Despite the strict relation between cell death and pathogen restriction, evidence that HR limits viral colonization remains controversial (Coll et al., 2011). Moreover, effectiveness and contribution of MP and other CiLV-C-encoded proteins in the movement of this virus remain unknown. However, regardless of whether or not cell death is responsible for restricting CiLV-C systemic movement, the presence of dead cells in association with ROS production are strong evidence of HR-like response elicitation in lesions surrounding the feeding sites of viruliferous mites.

In line with the integration of the RNA silencing with the classic frame of resistance by PAMP-triggered immunity (PTI) and effector-triggered immunity (ETI) (Pumplin and Voinnet, 2013; Sansregret et al., 2013), it is tempting to speculate that the host plants could recognize CiLV-C during activation or suppression of RNA silencing and summon a classic defense response. Recognition of CiLV-C could be from dsRNA produced by virus replication or secondary siRNA derived from RISC, triggering PTI defenses. Otherwise, plant could trigger an ETI response through R-mediated recognition of the putative VSR. In both cases, activation of the SA may convey to an HR-like response, restricting the virus near the inoculation site by the mite vector. Altogether, sequences of facts here reported during Arabidopsis/CiLV-C interaction, e.g., expression of HR-related genes, activation of effective defense pathways against viral infections, ROS burst sustained along the infection, localized cell death and pathogen restriction at the infection site, suggest that CiLV-C symptoms are the outcome of a HR-like resistance. Consequently, citrus leprosis should be considered the result of an incompatible rather than a compatible interaction.

Finally, responses of sweet orange to the *Brevipalpus* feeding and CiLV-C infection mirrored those detected in Arabidopsis. Our analysis revealed the activation of SA- and JA/ET-dependent defenses against mite feeding. Upon viral infection on viruliferous mites-infected plants, we observed induction of SA pathway and AGO2-mediated RNA silencing and reduction of the JA/ET defenses. However, the defense response was not established at 6 hai, which indicates a later response of *C. sinensis*. Time of symptoms appearance is likely an indicative of this phenomenon: lesions developed from 17 to 25 dai on the woody perennial *C. sinensis*, while in the herbaceous Arabidopsis this period was reduced to 7 to 10 dai. Although with a slower progression, the natural host developed a similar pattern of response than Arabidopsis, which validates this species as a model to be used in interaction studies of citrus leprosis disease.

**Figure 7:**
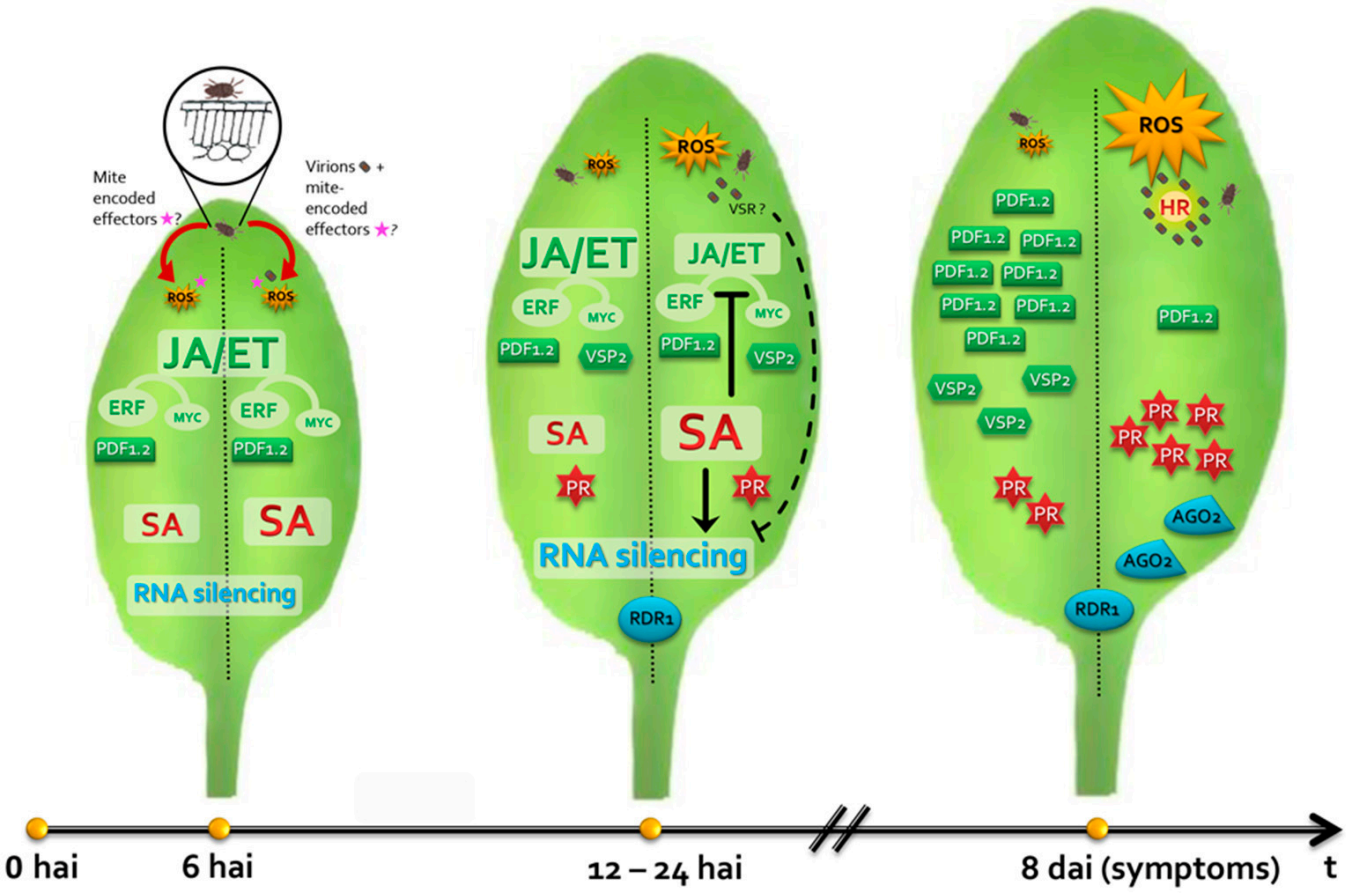
**Model representing the interaction of *Arabidopsis thaliana* plants/*Brevipalpus* mites/CiLV-C**. Left and right leaf halves show hallmarks during the plant interaction with non-viruliferous and viruliferous mites, respectively. During the feeding of non-viruliferous mites, saliva and putative mite-encoded effectors are delivered into the mesophyll cells targeted by the mite's stylet. Reactive oxygen species (ROS) burst, localized cell death and SA and JA/ET pathways are triggered. Mite induces the expression of the ERF-branch responsive gene *PDF1.2* rather than the MYC-branch responsive gene *VSP2*, which might restrain plant response against herbivores. In plant infested by CiLV-C viruliferous mites, virions reach mesophyll cells mixed with the salivary flow. CiLV-C multiplies in the initially infected cell and locally moves to the neighboring cells. Mite feeding and the increasing viral loads generate a stronger stimulus which intensifies the ROS production, the number of dead cells around the mite feeding site and the SA-mediated response. Increased activity of the SA pathway likely promotes the inhibition of the JA/ET pathways, leading the downregulation of *PDF1.2* and *VSP2*, which probably improve the mite performance. Both non-viruliferous and viruliferous mite infestations induce expression profile changes in the core genes of the gene silencing mechanism. Enhanced SA signaling activity in viral presence may contribute to higher *RDR1* expression. A putative virus suppressor of RNA silencing (VSR) may target and inactivate AGO1, leading to the up-regulation of *AGO2*, which addresses a second antiviral defense line of the RNA silencing. CiLV-C remains restricted at cells surrounding the inoculation site and chlorosis symptoms develop seven to 10 days after inoculation, probably as a result of a hypersensitive-like response (HR) as consequence of an incompatible interaction.

With the results presented here, we propose the first model of Arabidopsis/*B. yothersi/*CiLV-C interaction (Figure 7). This model represents a starting point to understand the processes leading to the development of citrus leprosis and, possibly, other diseases caused by non-systemic viruses also transmitted by these vectors. Future studies shall aim at complementing the model especially with regards to the lacking components and to the contribution of each hormonal pathway to such conspicuous interaction.

## Materials and methods

### Plant material

Seeds of the *Arabidopsis thaliana* ecotype Columbia (Col-0) were obtained from the Arabidopsis Biological Resource Center (ABRC). Seeds were sown in sterilized soil in 100 mL pots and incubated during 4 days at 4°C in a dark chamber. After this, plantlets were transferred to a controlled growth chamber (Adaptis AR A1000, Conviron, Winnipeg, Canada) set at 22 ± 2°C and with a 12 h light/dark cycle where they were kept throughout the experiments. *Citrus sinensis* L. Osbeck cv. Pera plants were grown from seeds under greenhouse conditions.

### Mite (*Brevipalpus yothersi*) rearing and infestation

A population of non-viruliferous mites was obtained from a single female collected from a citrus orchard in the State of Bahia, Brazil, and further confirmed as *B. yothersi* using phase contrast microscopy as reported elsewhere (Beard et al., 2015). Mites were reared onto unripe fruits of leprosis-immune “Tahiti” acid lime (*Citrus latifolia* Tanaka). All fruits were previously cleaned, dried and partially submerged in liquid paraffin to prevent desiccation. Mites were transferred to an area of approximately 4 cm in diameter of the fruit surrounded by a barrier of the pest adhesive Biostop gum (Biocontrole, Indaiatuba, Brazil) prepared with a wet mixture of wheat flour, plaster, and fine sand (1:1:2) (Rodrigues et al., 2007). Viruliferous mites were obtained by rearing the non-viruliferous mites from “Tahiti” acid lime as described above on sweet oranges fruits collected from a citrus grove with high incidence of leprosis caused by the CiLV-C strain SJP (Ramos-González et al., 2016) in São José do Rio Preto, State of São Paulo, Brazil. Mites from both viruliferous and non-viruliferous populations were reared for several generations and periodically evaluated for the presence of CiLV-C by RT-PCR using primer pairs designed for a region within the CiLV-C *MP* gene (Locali et al., 2003).

### Assessment of CiLV-C inoculation access period by viruliferous *B. yothersi* mites

To define the period needed to achieve 100% of CiLV-C infection of plants after infestation with viruliferous *B. yothersi* mites, five viruliferous mites were transferred to each of 10 Arabidopsis plants, where they were kept for 4 or 6 h. After the inoculation period, mites were removed using a small brush and plants were maintained in a controlled chamber until the development of symptoms. As positive controls, 10 plants were kept infested with viruliferous mites throughout the experiment. The inoculation periods were predetermined considering available data of CiLV-C infection of common bean (*Phaseolus vulgaris*) plants using viruliferous *B. yothersi* (Garita, 2013). Plants were evaluated daily for symptoms and CiLV-C infection was evaluated by RT-PCR (Locali et al., 2003) at the end of the experiment.

### Time-course gene expression analysis on arabidopsis

A time course gene expression analysis was conducted on plants infested with viruliferous or non-viruliferous mites and on non-infested control plants at 6, 12, and 24 h after infestation (hai) and at 8 days after infestation (dai), when symptoms were visible. Four-week-old *A. thaliana* Col-0 plants were grouped in sets of 20 individuals and were assigned to each treatment. For infestation, five (viruliferous or non-viruliferous) mites were transferred to each of three expanded rosette leaves per plant using a small brush and a stereoscopic microscope. Infested or control leaves were collected at each time-point; leaf samples from two plants were pooled, totaling 10 biological replicates per treatment per time point. Once collected, leaves were flash-frozen in liquid N_2_ and stored at −80°C until RNA extraction.

### Time-course gene expression analysis on *C. sinensis*

The time course experiment on sweet orange (*C. sinensis*) seedlings was established with the same infestation treatments and two time points after infestation: 6 hai and 25 dai, corresponding to 100% inoculate and 100% symptomatic plants, respectively. Selection of 6 hai as the first time for evaluation was based on previous results of inoculation access period in Arabidopsis obtained in this work, and those described elsewhere using common bean plants (Garita et al., 2013). Twenty plants per treatment were assayed. Fifteen mites were transferred to one leaf per plant, which was previously coated with a wet mixture of wheat flour, plaster and fine sand (1:1:2) as described above (Figure 1) (Rodrigues et al., 2007). Each collected leaf represented an independent biological replicate, totaling 20 biological replicates per treatment per time point. Once collected, leaves were flash-frozen in liquid N_2_ and stored at −80°C until RNA extraction.

### RNA extraction and cDNA synthesis

Plant RNA was purified from approximately 100 mg of leaves using the RNeasy Plant Mini Kit (Qiagen, Venlo, Netherlands). Residual plant DNA was removed by RNAse free DNAse (Qiagen, Venlo, Netherlands) during RNA extraction. RNA quantification and A_260_/A_280_ ratios were estimated using the NanoDrop ND-8000 micro-spectrophotometer (Thermo Scientific, Waltham, MA, USA). RNA integrity was evaluated in 1.2% agarose gels and the removal of genomic DNA was confirmed by reverse transcription (RT-) PCR assays using the RNA as template. cDNA corresponding to each sample (500 ng of total RNA) was generated using the RevertAid H Minus First Strand cDNA Synthesis Kit (Thermo Scientific, Waltham, MA, USA) as described by the manufacturer. cDNA solutions were diluted 25-fold in RNAse-free water for subsequent analysis by qPCR.

### CiLV-C detection and quantification

CiLV-C loads were assessed by RT-qPCR using a TaqMan® probe complementary to the viral *p29* ORF (G.D. Arena, P.L. Ramos-González, M.A. Machado, J. Freitas-Astúa, *unpublished data*). Reaction mixes were prepared as recommended by the TaqMan® Fast Universal PCR MasterMix 2X kit manufacturer (Thermo Scientific, Waltham, MA, USA) and the amplifications were carried out in a 7500 Fast Real-Time PCR System device (Thermo Scientific, Waltham, MA, USA). Each sample was analyzed in triplicate and three template-free controls were performed for each primer pair. Cq-values were compared with a standard curve to determine absolute quantities of CiLV-C *p29* molecules. Absolute *p29* quantities in infected *A. thaliana* and *C. sinensis* were normalized using the expression levels of the species-specific *SAND* genes as references. Average of the *p29* quantities of each time point were statistically compared with one-way ANOVA and Tukey's HSD (honest significant difference) tests (α < 0.05).

### Reference genes evaluation

Six candidate genes were selected for expression stability analyses based on previous data obtained from *A. thaliana* subjected to different stresses (Czechowski et al., 2005) or virus infection (Lilly et al., 2011) or considering the expression profile of their *Citrus* spp. homolog during the infection with CiLV-C (Mafra et al., 2012). The candidates *EF1A, F-BOX, GAPDH, PPR, SAND*, and *TIP41* were amplified using the primer pairs listed in Supplementary Table 1. Expression levels were assessed by RT-qPCR in Arabidopsis plants infested by either viruliferous or non-viruliferous mites and in non-infested plants. Cq-values and relative quantities were determined as described below for gene expression analysis. Cq-values were imported into geNorm software (Vandesompele et al., 2002) for stability analysis and further selection of the reference genes with the lowest *M*-values.

### Gene expression analysis

Transcript levels of defense-related genes were assessed by RT-qPCR. All primer sequences were obtained from the literature (Supplementary Table 1) and validated by PCR and RT-qPCR. RT-qPCR mixes were prepared for a final volume of 25 uL with 10 uL of GoTaq qPCR Master Mix (Promega, Madison, WI, USA), 120 or 150 nM of each gene-specific primer pair and 3 uL of the diluted cDNA. Amplifications were performed in a 7500 Fast Real-Time PCR System (Thermo Scientific, Waltham, MA, USA) device, using the standard thermal profile: 95°C for 20 s followed by 40 cycles of 95°C for 3 s and 60°C for 30 s. Each sample was analyzed in triplicate. To confirm the absence of genomic DNA and unspecific reactions, the dissociation curves of each reaction was inspected and three template-free controls were included for each primer pair. After this, primer efficiency and quantification cycle values were determined for individual RT-qPCR using the algorithm of the Real-time PCR Miner (Zhao and Fernald, 2005). Gene expression analyses were performed according the ΔCq model using multiple reference genes (Hellemans et al., 2007). The efficiency value (E) of each primer pair was estimated as the arithmetic mean of values from all samples. The Cq-value of each sample, expressed as the mean of the three technical replicates, was converted into relative quantities (RQ) using the function RQ = E^ΔCq^, where ΔCq is the difference between the lowest Cq-value across all samples for the evaluated gene and the Cq-value of a given sample. A normalization factor (NF) for each sample was calculated by the geometric mean of the RQ-values of the three reference genes. Normalized-relative quantity (NRQ) of each sample was calculated as the ratio of the sample RQ and the appropriate NF. Individual fold change values were determined by dividing the sample NRQ by the mean NRQ of samples of the calibrator, that is, non-infested control plants; this procedure renders a mean fold change value of 1 for the set of plants in the non-infested treatment. For statistical analysis, one-way ANOVA and Tukey's HSD test (α < 0.05) were used to compare the mean expression levels of the treatments. Statistical analysis was individually done for each gene at each time-point. For the *EDS5* and *NHL10* genes, the number of transcripts in mite-free plants was lower than the detection limit of the assay. In those cases, Cq-values were not determined and the data of mite-infested plants were considered as highly significant.

### Histochemical detection of H_2_O_2_ and cell death

Histochemical analyses of Arabidopsis plants were carried out in a time course manner with the same infestation treatments and time points of the gene expression analysis. Ten plants per treatment combination were assayed. Five mites were transferred to each of two expanded rosette leaves per plant. Each infested leaf of a plant was assayed for H_2_O_2_ production or cell death. Dead cells were visualized by staining with trypan blue (Martinez de Ilarduya et al., 2003). Infested or control leaves were boiled in the presence of lactophenol-trypan blue for 2 min and incubated overnight at room temperature. Excess dye was removed with chloral hydrate (2.5 g/mL) for 4 days. H_2_O_2_ was detected by staining with DAB (Martinez de Ilarduya et al., 2003). Leaves were vacuum infiltrated for seconds and incubated for 5 h in the dark to allow the infiltration of DAB. Tissues were boiled in a mix of ethanol:acetic acid (3:1) for 15 min. Samples were examined under bright-field light using an Olympus MVX10 (Olympus, Tokyo, Japan) microscope and images were capture with an Olympus DP71 (Olympus, Tokyo, Japan) camera. Brownish areas were measured with the QUANT software (Vale et al., 2003).

### Biology behavior of viruliferous and non-viruliferous mites in wild type arabidopsis plants

Two groups of 15 and 11 healthy Arabidopsis Col-0 plants were infested with CiLV-C viruliferous or non-viruliferous *Brevipalpus yothersi* mites, respectively. Five mites were transferred to each of three expanded rosette leaves of each plant (primary infestation), completing fifteen mites per plant. Sources of mites and their manipulation were described above. After 13 days of infestation, plant leaves were carefully detached, and the number of *Brevipalpus* eggs and adults were counted. The number of eggs and mites found in the primary infested leaves were compared to those found in the rest of the leaves (secondary infestation) using the Mann Whitney non-parametric test implemented in R. Furthermore, the same data were compared using t-test, with similar results. For the parametric test, data were normalized following ln(x) transformation. Presence or absence of CiLV-C in leaf samples was confirmed by RT-qPCR as described above.

## Author contributions

GA, PR, and JF conceived and designed the experiments; GA, PR, and MN performed the experiments; GA, PR, MR, LC, and JF analyzed the data; EK, MR, MM, and JF contributed with reagents/materials/analysis tools; GA, PR, and JF wrote the paper.

## Funding

The authors are grateful to CNPq and FAPESP for scholarships and research grants associated to this work (FAPESP 2012/18771-0, 2014/00366-8, 2008/57909-2 and 2014/08458-9; CNPq 573848/08-4 and 375843/2012-4).

### Conflict of interest statement

The authors declare that the research was conducted in the absence of any commercial or financial relationships that could be construed as a potential conflict of interest.
