# Supplementary material for: Citrus leprosis virus C Infection Results in Hypersensitive-Like Response, Suppression of the JA/ET Plant Defense Pathway and Promotion of the Colonization of Its Mite Vector
Source: Front Plant Sci. 2016 Nov 25;7:1757. doi: 10.3389/fpls.2016.01757 (PMC5122717; doi:10.3389/fpls.2016.01757)
Supplement: Supplementary file 1 [file Table1.docx]

**Supplementary Table 1**. Selected genes and corresponding primer pairs of *Arabidopsis thaliana* and *Citrus sinensis* used for gene expression analyses by RT-qPCR.

| Gene | Name | Locus | Sequences (5´-3´) | Reference |
| --- | --- | --- | --- | --- |
| *Arabidopsis thaliana* | | | | |
| *AGO1* | *Argonaute 1* | AT1G48410 | F:TCTACAGGGATGGAGTCAGTGAGGG | (Aelbrecht et al., 2006) |
|  |  |  | R:AGCCTCGTGTGATGACGCTTCTG |  |
| *AGO2* | *Argonaute 2* | AT1G31280 | F:GATGGAGCCTTGAGTACACGACCT | (Barrero et al., 2010) |
|  |  |  | R:AATGGACCGAGCGGTGGTGAAATC |  |
| *DCL2* | *Dicer-like 2* | AT3G03300 | F:AATCTCTAGCAGGCGCGATA | (Gutierrez et al., 2009) |
|  |  |  | R:TCACAGGATGCAACTTGACAG |  |
| *DCL4* | *Dicer-like 4* | AT5G20320 | F:GGCATCCGAAATGAAGCTAA | (Gutierrez et al., 2009) |
|  |  |  | R:CTTTCACTATTCCCGCCAAA |  |
| *EDS5* | *Enhanced disease susceptibility 5* | AT4G39030 | F:CATCAGGTGATGGCTCAGAC | (Wang et al., 2010) |
|  |  |  | R:ACTAATCCAAGCGTGGCTCC |  |
| *EF1α* | *Elongation factor 1-α* | AT5G60390 | F:CACCACTGGAGGTTTTGAGG | (Lilly et al., 2011) |
|  |  |  | R:TGGAGTATTTGGGGGTGGT |  |
| *EIN2* | *Ethylene insensitive 2* | AT5G03280 | F:TCATGGCGATTTCGAAGGTCTG | (Brotman et al., 2012) |
|  |  |  | R:AGGAAGCCCTAACAGAGCAACC |  |
| *ETR1* | *Ethylene response 1* | AT1G66340 | F:TCCAAGACCATCGCTCTCAATCCA | (Czechowski et al., 2004) |
|  |  |  | R:CCTCGCCATCTCCAAGAGGTTTGT |  |
| *FBOX* | *F-box family protein* | AT5G15710 | F:GGCTGAGAGGTTCGAGTGTT | (Lilly et al., 2011) |
|  |  |  | R:GGCTGTTGCATGACTGAAGA |  |
| *GAPDH* | *Glyceraldehyde-3-phosphate dehydrogenase* | AT1G13440 | F:TTGGTGACAACAGGTCAAGCA | (Brotman et al., 2012) |
|  |  |  | R:AAACTTGTCGCTCAATGCAATC |  |
| *HEN1* | *Hua enhancer 1* | AT4G20910 | F:TTCAATCAATGGGCATCAAA | (Gutierrez et al., 2009) |
|  |  |  | R:ACTTCACCAGACCCACCAAC |  |
| *ICS1* | *Isochorismate synthase 1* | AT1G74710 | F:GAGACTTACGAAGGAAGATGATGAG | (Chen et al., 2009) |
|  |  |  | R:TGATCCCGACTGCAAATTCACTCTC |  |
| *JAR1* | *Jasmonate resistant 1* | AT2G46370 | F:GTCATCACAAATTACGCAGGGTTG | (Brotman et al., 2012) |
|  |  |  | R:TCTCTTTCGGTGTTCTTGTCGATG |  |
| *MYC2* | *Jasmonate insensitive 1* | AT1G32640 | F:AGCAACGTTTACAAGCTTTGATTG | (Anderson et al., 2004) |
|  |  |  | R:TCATACGACGGTTGCCAGAA |  |
| *NHL10* | *NDR1/HIN1-like 10* | At2g35980 | F:TTCCTGTCCGTAACCCAAAC | (Boudsocq et al., 2010) |
|  |  |  | R:CCCTCGTAGTAGGCATGAGC |  |
| *NPR1* | *Nonexpresser of PR genes 1* | AT1G64280 | F:GTCGGTGAGACTCTTGCCTC | (Kim et al., 2010) |
|  |  |  | R:CGAAGAGCGAAACTATATGACG |  |
| *PDF1.2* | *Plant defensin 1.2* | AT5G44420 | F:CTTGTTCTCTTTGCTGCTTTCGAC | (López et al., 2011) |
|  |  |  | R:TTGGCTCCTTCAAGGTTAATGCAC |  |
| *PPR* | *Pentatricopeptide repeat superfamily protein* | AT5G55840 | F:AAGACAGTGAAGGTGCAACCTTACT | (Czechowski et al., 2005) |
|  |  |  | R:AGTTTTTGAGTTGTATTTGTCAGAGAAAG |  |
| *PR1* | *Pathogenesis-related gene 1* | AT2G14610 | F:GTGCCAAAGTGAGGTGTAACAA | (Lindermayr et al., 2010) |
|  |  |  | R:CGTGTGTATGCATGATCACATC |  |
| *PR5* | *Pathogenesis-related gene 5* | AT1G75040 | F:ATGTGAGCCTCGTAGATGGTTAC | (Lindermayr et al., 2010) |
|  |  |  | R:GATCCATGACCTTAAGCATGTCG |  |
| *RBOHD* | *Respiratoy burst oxidase homologue D* | AT5G47910 | F:CTAGCTTTGGATTTTTCTCGA | (Li et al., 2012) |
|  |  |  | R:GTAACCAACAAAACGGTAGGG |  |
| *RDR1* | *RNA-dependent RNA polymerase 1* | AT1G14790 | F:CGCCAAGAACGTCTGAACCA | (Lilly et al., 2011) |
|  |  |  | R:TTCGCGATGATCCCTAAACT |  |
| *RDR6* | *RNA-dependent RNA polymerase 6* | AT3G49500 | F:AGAAACTCATCCCTCCCAACAG | (Aelbrecht et al., 2006) |
|  |  |  | R:CCAACTGCTCATTCGCCAAG |  |
| *SAND* | *SAND family protein* | AT2G28390 | F:AACTCTATGCAGCATTTGATCCACT | (Czechowski et al., 2005) |
|  |  |  | R:TGATTGCATATCTTTATCGCCATC |  |
| *TIP41* | *TIP41-like family protein* | AT4G34270 | F:GTGAAAACTGTTGGAGAGAAGCAA | (Czechowski et al., 2005) |
|  |  |  | R:TCAACTGGATACCCTTTCGCA |  |
| *TGA3* | *TGA1A-related gene 3* | AT1G22070 | F:TCTCTTGAAGCAGAACCGTCGAG | (Brotman et al., 2012) |
|  |  |  | R:TCTAACTGTTGAACATGGGCCTTC |  |
| *VSP2* | *Vegetative storage protein 2* | AT5G24770 | F:ACGCAAAATATGGATACGGAACAG | (Bischoff et al., 2009) |
|  |  |  | R:CGGTCCCTAACCACAACCAGTA |  |
| *WRKY70* | *WRKY DNA-binding protein 70* | AT3G56400 | F:GGAAGAAGACAATCCTCATCGT | (Von Saint Paul et al., 2011) |
|  |  |  | R:CGTTTTCCCATTGACGTAACT |  |
| *Citrus sinensis* | | | | |
| *AGO2* | *Argonaute 2* | AT1G48410 | F:ATGTTATGGGATGAGCACG | This work |
|  |  |  | R:CAAGGTCAGCATAGTACACAGG |  |
| *MYC2* | *Jasmonate insensitive 1* | AT1G32640 | F:CGATAACGGCTCTGTAATGG | This work |
|  |  |  | R:GTTGTTGTTGCTGTTGTTGG |  |
| *PR1* | *Pathogenesis-related gene 1* | AT2G14610 | F: AAATGTGGGTGAATGAGAAAGC | (Mafra et al., 2013) |
|  |  |  | R: ATTATTGTTGCACGTCACCTTG |  |
| *SAND* | *SAND family protein* | AT2G28390 | F: AGGTTGTCAGCCTTGTTGGT | (Mafra et al., 2012) |
|  |  |  | R: GCCGTGGGATTATATCTTGG |  |
| *WRKY70* | *WRKY DNA-binding protein 70* | AT3G56400 | F: GTCCTTCGCCGCCGGTCTCT | (Mafra et al., 2013) |
|  |  |  | R: TCAGCAGCAGCAGGCGAATTCTT |  |

**Supplementary Table 2.** Number of *Brevipalpus yothersi* eggs and adult mites (mean ± standard error) in *Arabidopsis thaliana* at 13 days after the infestation with non-viruliferous (NV) and CiLV-C viruliferous (V) mites.

|  | **Primarily infested leaves^a^** | **Secondary infested leaves^b^** | **Total** | **Replicates^c^** |
| --- | --- | --- | --- | --- |
| V-eggs | 39.9 ± 5.9** | 11.4 ± 3.1 | 51.3 ± 6.7 | 15 |
| NV-eggs | 30.1 ± 6.4 | 21.6 ± 2.8 | 51.7 ± 6.9 | 11 |
| V-mites | 4.9 ± 1.0 | 3.4 ± 0.8 | 8.3 ± 0.7** | 15 |
| NV-mites | 1.7 ± 0.6 | 3.9 ± 0.7* | 5.6 ± 0.6 | 11 |

^a^Leaves where mites were originally deposited.

^b^Contiguous leaves infested due to mite migration.

^c^Total number of plants assessed under each treatment.

Number of eggs and mites found in the primarily infested leaves were compared with those found in secondary infested leaves. Likewise, total number of eggs and mites were compared between plants infested with non-viruliferous (NV) and viruliferous (V) mites. Statistically significant differences at *p-values* < 0.05 (*) or < 0.01 (**) using both non-parametric Mann Whitney test on the original data and parametric t-test on the ln-transformed data are indicated.
